# The Pharmacokinetics of Raloxifene and Its Interaction with Apigenin in Rat

**DOI:** 10.3390/molecules15118478

**Published:** 2010-11-18

**Authors:** Yan Chen, Xiaobin Jia, Jian Chen, Jinyan Wang, Ming Hu

**Affiliations:** 1 Key Laboratory of New Drug Delivery System of Chinese Materia Medica, Jiangsu Provincial Academy of Chinese Medicine, 100 Shizi Road, Nanjing 210028, China; E-Mails: ychen202@hotmail.com (Y.C.); blacker2000@126.com (J.C.); wwind924@yahoo.com.cn (J.W.); 2 Department of Pharmacological and Pharmaceutical Sciences, College of Pharmacy, University of Houston, 1441 Moursund Street, Houston, TX 77030, USA; E-Mail: mhu@uh.edu (M.H)

**Keywords:** raloxifene, apigenin, pharmacokinetics, interaction, flavonoid, rats

## Abstract

*Purpose*: Raloxifene is a selective estrogen receptor modulator which is structurally similar to tamoxifen. As flavonoids can interact with raloxifene *in vitro*, we evaluated the *in vivo* pharmacokinetics of raloxifene in rats when co-administered with apigenin. *Methods*: The pharmacokinetics of raloxifene in the absence or presence of apigenin was investigated in rats after different dosage regimens. The plasma concentrations before and after enzymatic hydrolysis were analyzed by HPLC, and the pharmacokinetic profiles of raloxifene administered alone and in combination with apigenin were compared. *Results*: Co-administration of apigenin with raloxifene in a 1:2 ratio by weight resulted in a 55% and 37% increase in the C_max_ and AUC of intact raloxifene, respectively. When equal proportions of raloxifene and apigenin (1:1) were administered, the C_max_ and AUC of intact raloxifene were increased by 173% and 97% respectively. This increase in intact raloxifene was not associated with an increase in total raloxifene (intact plus conjugated raloxifene) because AUC and C_max_ of total raloxifene when administered alone or in combination with apigenin were found to be similar. The results indicated that apigenin inhibited the glucuronidation and sulfation of raloxifene in the intestine bringing about an increased bioavailability of the drug. *Conclusions*: The results showed that apigenin decreased the first-pass metabolism of raloxifene but did not increase its absorption from the gastrointestinal tract.

## 1. Introduction

Raloxifene is a selective estrogen receptor modulator (SERM) that produces both estrogen-agonistic effects on bone and lipid metabolism and estrogen-antagonistic effects on the uterine endometrium and breast tissue. Because of its tissue selectivity, fewer side effects may be observed with the use of raloxifene as compared to first generation SERMs (e.g., tamoxifen). Some of the beneficial estrogenic effects of raloxifene include increasing bone mineral density and decreasing total and low-density lipoprotein cholesterol levels [1,2,3]. Raloxifene has already been approved by the U.S. Food and Drug Administration for the prevention of osteoporosis and breast cancer in high risk patients.

The pharmacokinetics of raloxifene has been studied. It was reported that the bioavailability of raloxifen in rats was approximately 39% [4]. Despite its rapid absorption in humans following oral administration, its bioavailability is significantly lower (2%) [5]. Raloxifene undergoes extensive biotransformation, but it does not appear to be metabolized by the cytochrome P450 pathway [6]. There is the potential metabolic interaction between raloxifene and drugs eliminated by the Phase II metabolism. The previous result indicated that raloxifene was extensively conjugated in rat intestine and liver microsomes, and that intestinal metabolism played a more important role in the presystemic metabolism than liver [7]. A raloxifene metabolic pathway similar to that in humans is observed in rats [8]. The major difference between metabolites in rats and humans is that the main metabolite in rats is raloxifene-6-β-glucuronide, whereas in humans it is the 4-β-glucuronide [7,9]. 

Apigenin is a common flavonoid presented in a variety of plants, vegetables, fruits and herbs, some of which are widely marketed as dietary and herbal supplements [10]. Therefore, a combination of flavonoids and raloxifene is expected in postmenopausal patients [11]. A concern arising from this possible combination is disposition interaction in the wall of the gut, because absorption and disposition of flavonoids are similar to that of raloxifene in enterocytes [12]. Specifically, *in vitro* studies of flavonoids showed that they were also extensively metabolized in the gut via phase II conjugation [13,14]. Additional studies showed that apigenin could reduce the conjugation of raloxifene *in vitro* [15]. However, there are no reports on the interaction between raloxifene and flavonoids such as apigenin *in vivo*. Therefore, our hypothesis is that coadministration of apigenin with raloxifene could increase the bioavailability of raloxifene by inhibiting PhaseⅡmetabolism of the drug, and then the mechanism of drug-drug interaction was explained.

## 2. Results and Discussion

**Figure 1:**
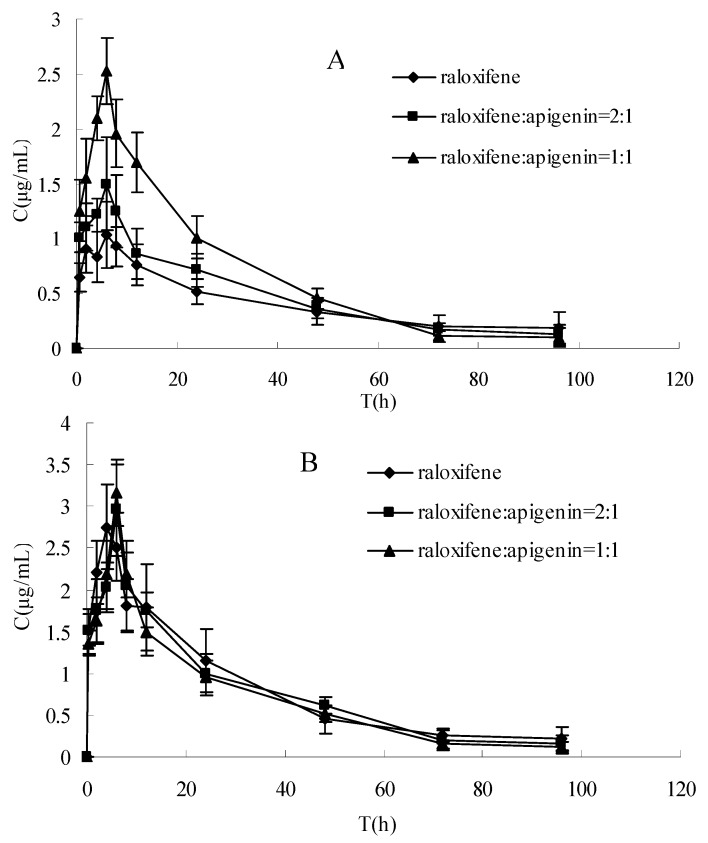
The rat pharmacokinetics profile of intact raloxifene of (A) before hydrolysis and (B) after hydrolysis of three groups in same graph. Each point represents the mean ± SD (*n* = 5).

**Figure 2:**
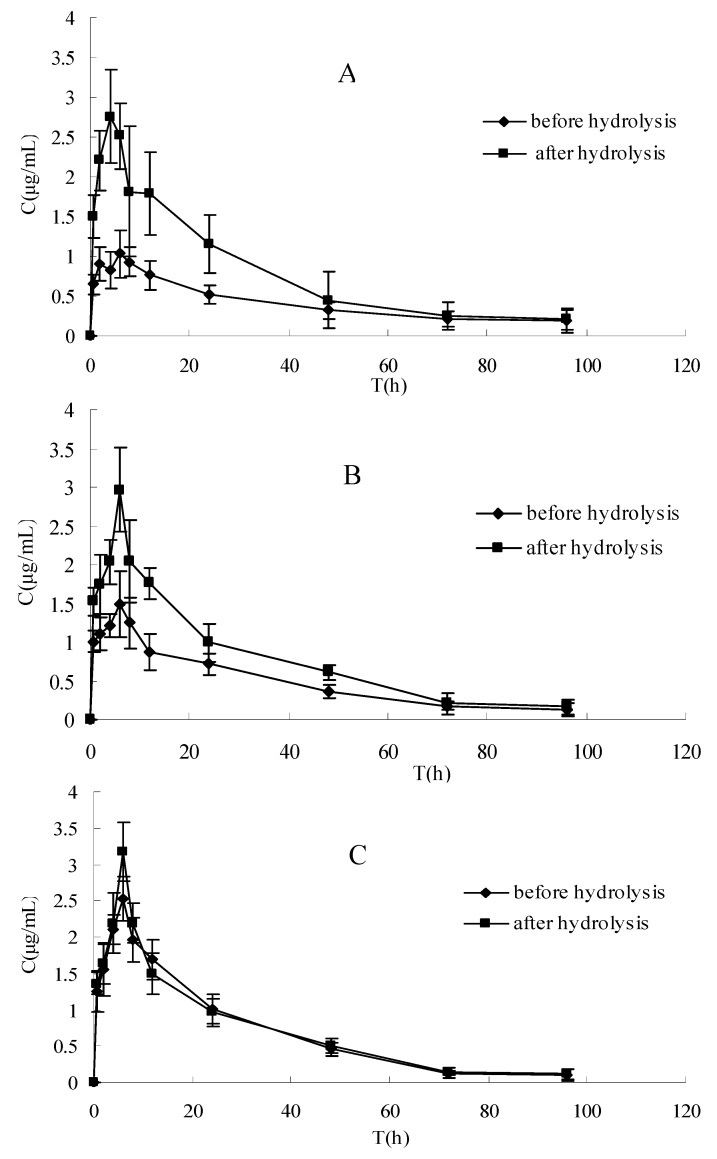
The pharmacokinetics profile of intact raloxifene before hydrolysis and total raloxifene after hydrolysis in different groups of rats. (A) Raloxifene alone, (B) raloxifene: apigenin = 2:1, (C) raloxifene:apigenin = 1:1. Each point represents the mean ± SD (*n* = 5).

The plasma concentration-time profiles of raloxifene in different dosage regimens before and after enzymatic hydrolysis are shown in Figure 1 and Figure 2. The pharmacokinetic parameters of raloxifene are summarized in Table 1. Before hydrolysis, the analysis of variance of t_1/2_ and t_max_ showed no differences (P > 0.05), while the AUC, C_max_ and CL had significant difference among the three groups after oral administration (P < 0.05). These results indicated that t_1/2_ and t_max_ did not vary with when apigenin was co-administered with raloxifene. The AUC increased with increasing apigenin dose. Furthermore, the C_max_ was also increased, which was consistent with the AUC. The CL decreased by apigenin. After hydrolysis, when plotted in the same graph (Figure 1), the time course for the total raloxifene (intact and released from sulfate conjugates and glucuronide conjugates) was nearly superimposed. This provided evidence that the pharmacokinetic data on total drug would be a good approximation for different groups. In Table 1, there was a significant difference in the AUC，C_max_ and CL (P < 0.05) between before and after hydrolysis in raloxifene alone and raloxifene: apigenin (2:1) group, whereas this phenomemon was not observed in the group of raloxifene: apigenin (1:1). In Figure 2, our data showed that the differences between the C_max_ of raloxifene before and after hydrolysis in various groups were significant. We found that the C_max_ values of raloxifene after hydrolysis were 2.91, 1.93 and1.16-fold higher than that before hydrolysis respectively in three groups. It was observed that raloxifene glucuronides/sulfates decreased when the amount of apigenin increased. This indicated that apigenin inhibited raloxifene glucuronidation/ sulfation, and resulted in the increased bioavailability of raloxifene in rats.

The aim of this study was to examine the effect of apigenin on the absorption and bioavailability of raloxifene in rats. The AUC of raloxifene increased by 37% (2:1) and 97% (1:1) for oral coadministration of apigenin after a single 10 mg·kg^−1^ intragastric administration. This result showed that the plasma concentration of the intact raloxifene increased gradually with spiked amount of apigenin before hydrolysis, whereas the relative exposure to the total raloxifene was similar without any statistical difference among three groups after hydrolysis. This indicated that the concomitant administration of apigenin with raloxifene resulted in an increased exposure to intact raloxifene dramatically rather than total raloxinfene absorbed dose (intact and conjugated drug) in plasma. Total exposure of circulating raloxifene (AUC value) after hydrolysis is 2.1 and 1.6-fold compared with intact drug before hydrolysis in raloxifene alone and raloxifene:apigenin (2:1) treatment, respectively, whereas it is not observed in raloxifene:apigenin (1:1) group. 

The results showed that the total drug absorbed into the blood was not affected by the co-administered apigenin; however, apigenin could improve the absorption fraction of intact raloxifene from intestine during the transport across monolayer enterocyte by competitively inhibiting the formation of raloxifene-glcuronide and raloxifene-sulfate in the gut.

Our study of the pharmacokinetic profile in rats of raloxifene alone and in combination with apigenin was based on much earlier *in vitro* studies. Reasons for the interaction between raloxifene and apigenin have been proposed and involve two mechanisms, namely extensive presystemic metabolism and excretion of the enterocyte efflux transporter (MRP, OAT, P-gp). The hypothesis that extensive metabolism of raloxifene in Caco-2 cell and in microsome had been reported [15]. The further discovery indicated that intestinal glucuronidation was the most important contributor to the presystemic clearance of raloxifene, whereas a minor amount of raloxifene was transformed into sulfate [7,15,16]. Therefore, extensive first pass metabolism of raloxifene after oral administration may diminish their transport into the systemic circulation. Apigenin, a flavone analog of estrogen, also has anticancer activities. Limited studies in rats suggested that it was also more extensively metabolized by glucuronidation in the gut, rather than in liver, although the structures of these conjugates have not been determined completely [17]. The pharmacokinetic parameters of raloxifene suggested that apigenin had the potential to increase the bioavailability of raloxifene when taken together [7]. Our results are consistent with the previous study. They also indicated that apigenin is a well substrate for various UGT (UDP-glucuronosyltransferase) isoforms, so it has the inhibitory effect on raloxifene [16]. Moreover, apigenin has an opportunity to competitively inhibit glucuronidation and sulfoconjugation of raloxifene in intestine microsomes *in vitro*. These studies suggested that the pharmacokinetics profile of raloxifene could be affected by apigenin coadministeration and the bioavailability of intact raloxifene could be enhanced via inhibition of phase II metabolism.

Recent studies indicated that efflux of raloxifene and its conjugated metabolites from Caco-2 monolayer [8,18]. Efflux transporters could be responsible for pumping out the intracellular intact drug and phase II metabolites. The efflux of raloxifene appeared to be mediated by MRP and P-glycoprotein, whereas the raloxifene conjugated metabolites were effluxed by MRP and OAT transporter. On the other hand, Hu and his coworker have reported the efflux mechanism responsible for the efflux of apigenin and its conjugated metabolites in enterocyte previously [19,20]. Because the efflux transporters in the gut are saturated, the interaction of raloxifene and apigenin may arise in the gut. The contribution of intestinal efflux transporter to the first-pass metabolism of orally ingested raloxifene and flavonoids has gained considerable interest in recent years. By the way, the increased drug exposure and bioavailability are little attributed to the interaction of renal excretion, because raloxifene is eliminated primarily in the feces directly or via bile and only negligible amounts appear in the urine (the urine excreted fraction is below 6% in rats and humans) [21].

Some absorbable raloxifene are metabolized to conjugate in enterocytes first, and then total raloxifene (intact and conjugated drug) will be effluxed to the lumen (AP) or transported into the liver via the portal vein, so the total raloxifene entering the systemic circulation include intact and conjugated drug. In our analysis condition, intact raloxifene were found exclusively circulating in the blood, but raloxifene glucuronides/sulfates was undetected in plasma in three group rats (data not shown), whereas the peak area of the raloxifene increased after incubation with *β*-glucuronidase and sulfatase at the same plasma sample at sequential time. In order to determine the total raloxifene exposure in plasma, we hydrolyzed the plasma and determined the total plasma concentration after hydrolysis. Because there were different binding sites of conjugates available for glucuronidation and sulfation, therefore, we used a mixed enzyme of glucuronidase/sulfatase to hydrolyze plasma to free all conjugated drug in our research. It cannot recognize binding site of conjugates and discriminate these bonds specially. The mechanism of apigenin increasing the bioavailability of raloxifene in rat is complicated. Our further study will focus on its mechanism by using Caco-2 cell model and *In Situ* Single-Pass Perfused Rat Intestinal Model. Besides, liver may be another important contributor to the clearance of raloxifene, so a study on the metabolism and drug-drug interaction in liver is also needed. 

## 3. Experimental

### 3.1. Chemicals

Raloxifene and apigenin were obtained by Dongsheng Chemical Co. Ltd (Zhejiang, China). The internal standard (IS), testosterone, was purchased from the Xianju Pharmaceutical Company. β-Glucuronidase (type B-1 from bovine liver), sulfatase (type H-1 from *Helix pomatia*), were purchased from Sigma Chemical Co. Ltd (St. Louis, Mo, USA). Acetonitrile was of HPLC grade. All the other chemicals were purchased from Nanjing Chemical Reagent (Nanjing, China). Purified water obtained via a Milli-Q system (Millipore, Bedford, MA, USA) was used throughout the experiments.

### 3.2. Animals

Wistar rats (300–350 g) were obtained from SLEK Lab Animal Center of Shanghai (Shanghai, China). Animals were kept on a 12 h light/dark cycle for a minimum of three days before experiments with free access to water and a standard diet. The studies were approved by the Animal Ethics Committee of Jiangsu Provincial Academy of Chinese Medicine. 

### 3.3. Animal study

Eighty rats (both sexes) were randomized into three experimental groups after a single oral administration. The three experimental groups included: 10 mg raloxifene alone (group 1), 10 mg raloxifene in combination with 5 mg·kg^−1^ apigenin (group 2), 10 mg raloxifene in combination with 10 mg·kg^−1^ apigenin (group 3). At 0, 0.5, 2, 4, 6, 8, 12, 24, 48, 72, 96 h after administration, plasma samples (0.2 mL) were collected and then divided into two aliquots to determine raloxifene concentration before and after hydrolysis with mixture of glucuronidases and sulfatases.

### 3.4. Enzymatic hydrolysis

One aliquot of plasma (100 uL) was analyzed for the total drug concentration after enzymatic hydrolysis with mixed glucuronidase with sulfatase (1,000 and 100 U, respectively) preparation at 37 °C for 3 h, which resulted in the complete release of the raloxifene. 

### 3.5. Sample preparation

The samples were prepared as follows: Briefly, testosterone (20 μL, 20 mg/mL) was added to plasma (100 μL) in a 1.5 mL tube and methanol (500 μL) was added to precipitate the protein. After vortex-mixing for 3 min, samples were separated by centrifugation at 14,000 rpm for 10 min, and then the supernatant was transferred to another tube and evaporated to dryness at 40 °C under a gentle stream of nitrogen. The residue was reconstituted in deionized water (200 μL) followed by vortex-mixing and centrifugation at 14,000 rpm for 15 min. Then, 40 μL of an aliquot of the supernatant was injected into the HPLC system. 

### 3.6. Method validation

**Figure 3:**
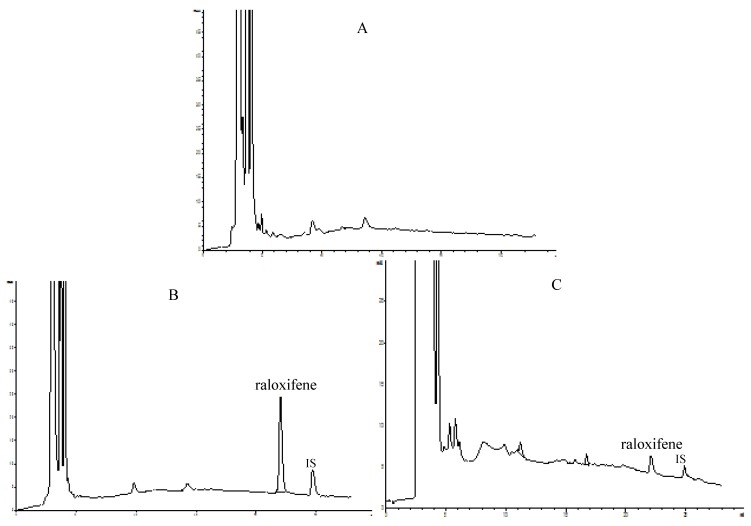
Representative chromatograms of raloxifene. (A) blank plasma; (B) plasma spiked with raloxifene and internal standard (testosterone); (C) plasma sample obtained 4 h after oral administration of raloxifene.

In the present study the concentrations of raloxifene in rat plasma were determined by an HPLC method performed using an Agilent 1200 system (Agilent Technologies, Palo Alto, CA, USA) equipped with an ODS column (5 μm, 250 mm × 4.6 mm). Elution of the raloxifene and internal standard (*i.e.*, testosterone) was carried out using a gradient of acetonitrile (A) and buffer solution (B, containing 0.04% phosphoric acid and 0.06% triethylamine, adjusted to pH 2.8 with triethylamine) at a flow-rate of 1.0 mL/min. The gradient elution of the mobile phase was 20% A in 0–3 min, 20–48.5% A in 3–22 min, 48.5% A in 22–25 min, 48.5–20% A in 25–26 min and 20% A 26–28 min. Detection was performed at a wavelength of 286 nm and a column temperature was 30 °C. The HPLC chromatograms of blank plasma, plasma spiked with raloxifene and internal standard and plasma sample obtained 4 h after oral administration of raloxifene are shown in Figure 3. 

The method was validated according to the requirement of biopharmaceutical analysis. The standard calibration curve for spiked rat plasma containing raloxifene was linear over the range 0.56–17.92μg·mL^−1^ with a correlation coefficient (*r*) > 0.9995, typical equations for the calibration curve was *Y* = 0.1219 *X* + 0.0259(where *X* is the plasma concentration of each analyte (μg·mL^−1^) and *Y* is the peak-area ratios of each analyte to IS); The lower limit of quantification (LLOQ) for determination of raloxifene was 0.56 μg·mL^−1^ and the limit of detection (LOD) was 0.17 μg·mL^−1^. The batch accuracy ranged from 91.5 to 112.0%, while within batch precision remained below 10.6%. Short-term stability showed that raloxifene was stable in plasma at least 16 h at room temperature, while long-term stability studies showed that raloxifene was stable in plasma for at least seven days when stored at −20 °C.

### 3.7. Pharmacokinetic analysis

The terminal half-life (t_1/2_) was determined by linear regression of the terminal elimination of the plasma concentration. The area under the plasma concentration-time curve from zero to the last measurable plasma concentration point (AUC_0-τ_) was calculated by the linear trapezoidal method. Extrapolation to time infinity (AUC_0-∞_) was calculated as follows: AUC_0-∞_ = AUC_0-τ_+ C_t_/k_e_. Maximum drug plasma concentration (*C*_max_) and time to reach the maximum concentration (*T*_max_) were taken directly from the observed data. Pharmacokinetic parameters were estimated using the DASS2.0 pharmacokinetic software, according to minimum of AIC principle. Data for multiple comparisons were analyzed by One-Way ANOVA with post hoc test and Student’s t-tests for double comparisons. The prior level p< 0.05 was considered significant. 

## 4. Conclusions

Concomitant oral administration of apigenin resulted in a significant and probably relevant increase in systemic raloxifene exposure after oral administration. The increased oral bioavailability of raloxifene was attributed to the inhibition of intestinal Phase II metabolism and efflux transporter function by oral apigenin. These results indicated that some flavones could enhance the absorption of drugs co-administered with low bioavailabilities, and thus enhance their efficacy. On the other side, the drug–drug interactions are common in clinical, and can cause toxic side effects if they increased drug levels reaching above toxic threshold [22]. We should aware the potential interaction of a drug with a narrow therapeutic index. As the interaction between raloxifene and apigenin is expected to occur in rat, patients treated concurrently with oral raloxifene and apigenin should be closely investigated.

## Figures and Tables

**Table 1:** The pharmacokinetic parameters of raloxifene after a single oral administration to rats before and after hydrolysis. (Mean ± S.D, n = 6).

Parameters	Dose					
	10 mg·kg^−1^ raloxifene		10 mg raloxifene in combination with 5 mg·kg^−1^ apigenin		10 mg raloxifene in combination with 10 mg·kg^−1^ apigenin	
	before	after	before	After	before	after
T_1/2_(h)	13.5 ± 2.4	12.6 ± 3.9	12.2 ± 2.6	16.1 ± 4.1	14.8 ± 3.2	13.1 ± 3.9
AUC(μg·h/mL)	31.7 ± 13.6	67.6 ± 21.2##	43.7 ± 13.8*	71.4 ± 22.4#	62.6 ± 14.2**	63.3 ± 25.9
CL/F(mL/h)	63.2 ± 22.1	29.6 ± 10.3##	45.8 ± 12.4*	28.0 ± 11.9#	31.9 ± 12.5**	31.6 ± 14.5
T_max_(h)	5.67 ± 0.83	5.33 ± 1.03	5.33 ± 1.03	5.33 ± 1.03	5.33 ± 1.03	5.67 ± 0.83
C_max_(μg/mL)	0.96 ± 0.31	2.79 ± 1.01##	1.49 ± 0.87*	2.88 ± 1.09#	2.63 ± 0.99**	3.05 ± 1.43

*compared with the raloxifene (10 mg·kg^−1^) alone group before hydrolysis (*p ＜ 0.05; **p ＜ 0.01); # compared with the pharmacokinetic parameters of raloxifene before hydrolysis and after hydrolysis in different group rats (#p ＜ 0.05; ##p ＜ 0.01).
